# First principles exploring the tunable electronic and optical features of silicane/γ-GeSe heterostructures for advanced electronic devices

**DOI:** 10.1039/d5na00181a

**Published:** 2025-05-08

**Authors:** Nguyen P. Q. Anh, Ho V. Cuu, Truong Tan, Chuong V. Nguyen, Nguyen N. Hieu

**Affiliations:** a Faculty of Engineering and Technology, Saigon University 273 An Duong Vuong Street, Ward 2, District 5 Ho Chi Minh City Vietnam; b Department of Materials Science and Engineering, Le Quy Don Technical University Hanoi 100000 Vietnam; c Institute of Research and Development, Duy Tan University Da Nang 550000 Vietnam hieunn@duytan.edu.vn; d Faculty of Natural Sciences, Duy Tan University Da Nang 550000 Vietnam

## Abstract

In this work, we explore the electronic and optical properties of the SiH/γ-GeSe heterostructure using first-principles calculations, emphasizing its remarkable tunability under applied electric fields. Our findings demonstrate that the SiH/γ-GeSe heterostructure exhibits stability, indicating its feasibility for future synthesis. The SiH/γ-GeSe exhibits type-I band alignment and an indirect band gap, with optical absorption characteristics revealing enhanced absorption in specific energy regions, highlighting its potential for advanced optoelectronic applications. Under the influence of electric fields, the SiH/γ-GeSe heterostructure transitions to type-II band alignment and switches to a direct band gap, which significantly improves charge separation and light absorption efficiency. These findings underscore the versatility of the SiH/γ-GeSe heterostructure, positioning it as a promising candidate for a wide range of electronic and optoelectronic applications.

## Abstract 2

In this work, we explore the electronic and optical properties of the SiH/γ-GeSe heterostructure using first-principles calculations, emphasizing its remarkable tunability under applied electric fields.

## Graphical abstract

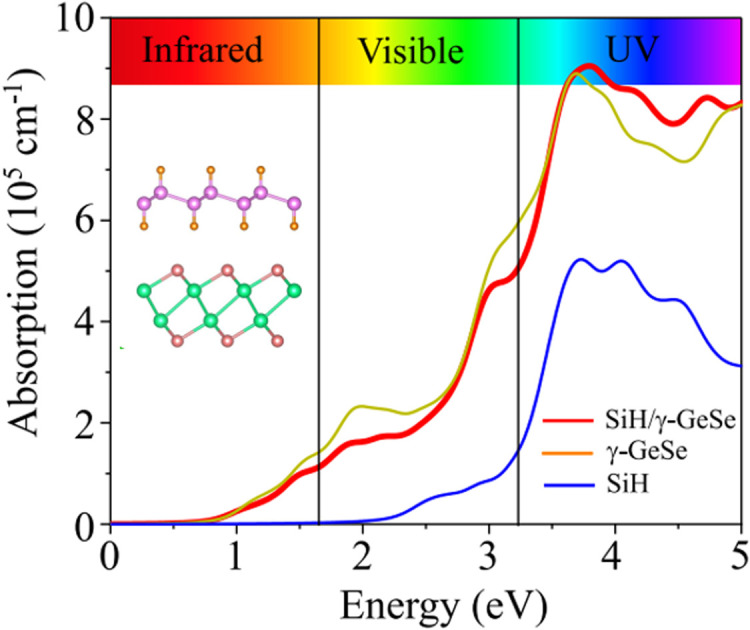

## 1 Introduction

The discovery of two-dimensional (2D) materials began with the isolation of graphene in 2004,^1^ a single layer of carbon atoms arranged in a hexagonal lattice. This breakthrough revolutionized materials science due to remarkable properties of graphene, including exceptional electrical conductivity and high carrier mobility.^2,3^ Since then, the field of 2D materials has expanded rapidly beyond graphene to include a wide variety of other materials, such as transition metal dichalcogenides (TMDs),^4,5^ black phosphorus,^6,7^ MXenes,^8,9^ MoSi_2_N_4_ (ref. 10) and Janus structures.^11,12^ Each new 2D material offers unique advantages, such as tunable band gaps in TMDs or high carrier mobility in phosphorene, leading to growing interest in heterostructures and multi-layer systems that combine the distinct properties of different 2D layers. The discovery of 2D materials has thus opened up a new frontier for research and technological innovation, offering unprecedented opportunities for the development of next-generation devices.

Recently, group-IV monochalcogenides have emerged as a particularly interesting class of materials due to their promising characteristics.^13,14^ One of the most recently discovered members of this family is γ-GeSe, a polymorph of GeSe, which has demonstrated remarkable potential for various applications. Recent experimental studies have successfully synthesized γ-GeSe monolayers *via* chemical vapor deposition (CVD), confirming its feasibility for large-scale production.^15^ γ-GeSe is a layered 2D material, structurally similar to other group-IV monochalcogenides, but with unique properties stemming from its polymorphic nature. Unlike the orthorhombic phase of GeSe, the γ-GeSe features a distinct atomic arrangement, which leads to different band structures and carrier mobility.^16^ The most notable feature of γ-GeSe is its Mexican-hat shaped band structure, which contributes to its fascinating electronic properties, such as a high density of states near the band edges that can be tuned through external factors such as strain,^17,18^ electric fields,^19^ and layer thickness.^20^

Similarly, silicane (SiH), a hydrogenated form of silicene,^21^ has emerged as an exciting new 2D material due to its unique combination of structural and electronic properties.^22,23^ Silicane is produced by the hydrogenation of silicene,^21^ a silicon-based analog of graphene, which itself has garnered interest for its compatibility with existing silicon-based technologies.^24,25^ Silicane retains hexagonal lattice of silicene but with significant alterations to its electronic properties due to the hydrogenation process. While silicene behaves like a semi-metal with a Dirac-like band structure, silicane transitions into a semiconductor with a tunable band gap, making it more suitable for applications where controlled electronic properties are crucial. This tunability, along with its inherent compatibility with silicon-based electronics, positions silicane as a highly versatile material for a wide range of applications, including field-effect transistors (FETs)^22^ and optoelectronic applications.^26^

In recent years, heterostructures formed from multiple 2D materials have opened up exciting new opportunities for engineering material properties that extend beyond the capabilities of single-layer systems.^27–29^ The 2D heterostructures can be synthesized in experiments through different approaches, such as molecular beam epitaxy^30,31^ and CVD.^32^ By carefully selecting and stacking materials with complementary characteristics, it becomes possible to design heterostructures with precisely tailored electronic properties, such as tunable band alignments and adjustable band gaps. 2D heterostructures between two semiconductors result in the formation of type-I, type-II, and type-III band alignments.^33–35^ Each type of band alignment is preferred for different applications, such as light-emitting devices, photodetectors, solar cells and tunneling field-effect transistors (TFETs). Among these, heterostructures based on silicane have garnered significant interest due to its unique properties and the compatibility with silicon-based technologies. Several studies have explored heterostructures incorporating silicane, demonstrating tunable electronic and optical properties through the combination of silicane with various 2D materials.^36–40^ Unfortunately, the combination of silicane with the novel γ-GeSe material has not yet been designed or systematically investigated. Given the distinct properties of γ-GeSe, including its Mexican-hat shaped band structure and high density of states near the band edges, a silicane/γ-GeSe heterostructure could offer novel electronic and optical properties that are highly tunable *via* external factors such as electric fields. The study of such a heterostructure would provide insights into new possibilities for material design.

## 2 Computational methods

All calculations in this study were conducted using first-principles methods within the framework of density functional theory (DFT), as implemented in the Quantum ESPRESSO package.^41^ We employed the Perdew–Burke–Ernzerhof (PBE) exchange–correlation functional to describe the electron–electron interactions.^42^ The plane-wave cutoff energy was set to 500 eV, and the Brillouin zone was sampled using a Monkhorst–Pack grid^43^ of 12 × 12 × 1. The weak van der Waals interactions were included using the DFT-D2 method of Grimme.^44^ The structural optimizations were performed until the forces on each atom were less than 0.01 eV Å^−1^ and the total energy convergence criterion was set to 10^−6^ eV. Phonon dispersion calculations were carried out using the density functional perturbation theory (DFPT)^45^ to confirm the dynamical stability of the heterostructure. *Ab Initio* Molecular Dynamics (AIMD) simulations were performed at 300 K for a duration of 8 ps using a time step of 1 fs.

## 3 Results and discussion

**Fig. 1:**
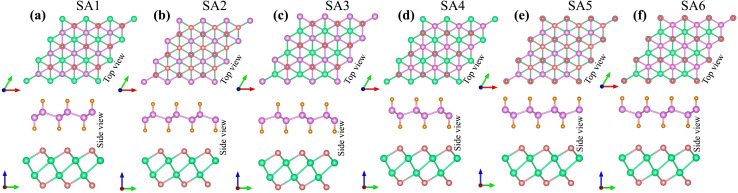
Different stacking arrangements of the SiH/γ-GeSe heterostructure of (a) SA1, (b) SA2, (c) SA3, (d) SA4, (e) SA5 and (f) SA6.

The lattice parameter of the SiH monolayer is 3.86 Å, whereas the lattice parameter of the γ-GeSe monolayer measures 3.80 Å. These values are consistent with previous reports,^15,46^ which supports the accuracy of our computational methods. Given the minimal difference in lattice parameters, the SiH/γ-GeSe heterostructure is constructed using a (1 × 1) unit cell for both the SiH and γ-GeSe monolayers to ensure compatibility. The lattice parameter for the SiH/γ-GeSe heterostructure is set at 3.83 Å, representing the average of the lattice parameters of the individual monolayers. The lattice mismatch in the SiH/γ-GeSe heterostructure is less than 1%, which is small and can be considered negligible since it does not significantly impact the intrinsic properties of each individual material. The different stacking arrangements (SA) of the SiH/γ-GeSe heterostructure are displayed in Fig. 1. We examine six distinct stacking configurations (SAs) of the SiH/γ-GeSe heterostructure. The interlayer spacing (*D*) between SiH and γ-GeSe layers are 2.11, 2.16, 2.16, 2.87, 2.15 and 2.9 Å, respectively, for SA1, SA2, SA3, SA4, SA5 and SA6. These values align well with the previous SiH-based heterostructures.^36,37,47–49^ The observed interlayer distances are greater than the sum of the covalent radii of hydrogen (0.31 Å) and selenium (1.20 Å) atoms, suggesting that the interactions between the SiH and GeSe layers are weak, consistent with vdW bonding rather than covalent or ionic interactions.

**Fig. 2:**
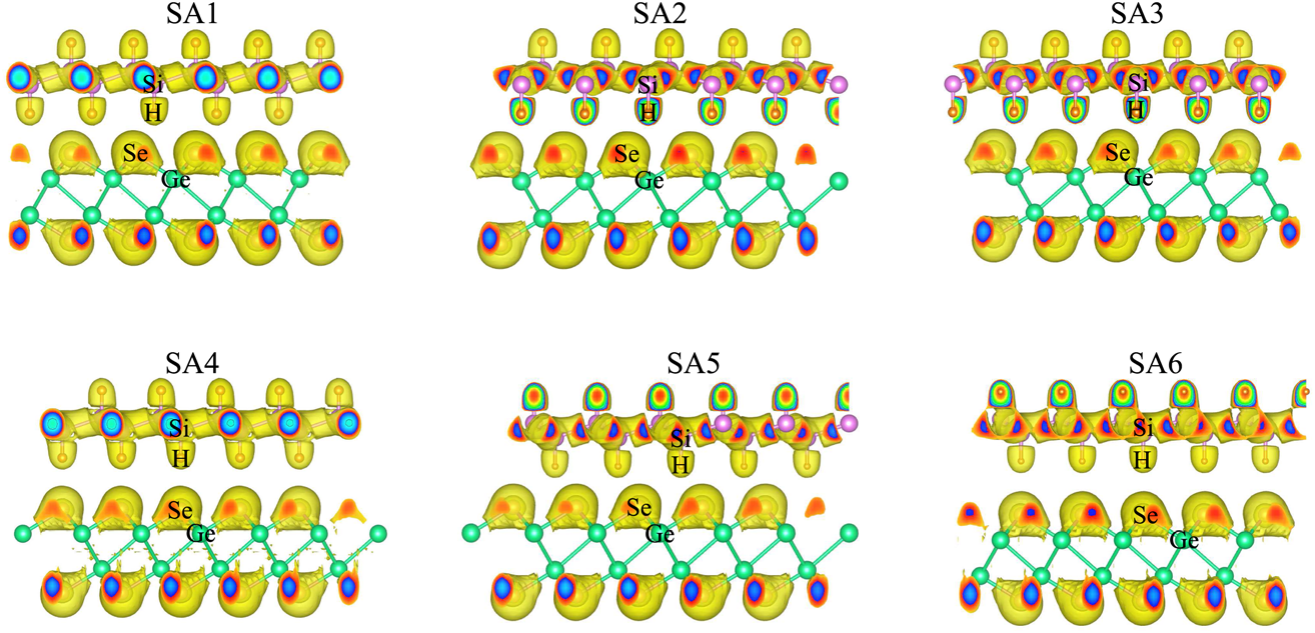
The visualized 3D electron localization functions of the SiH/γ-GeSe heterostructure in all six configurations.

The binding energy (*E*_b_) is calculated to assess the stability of the heterostructure using the following equation:1
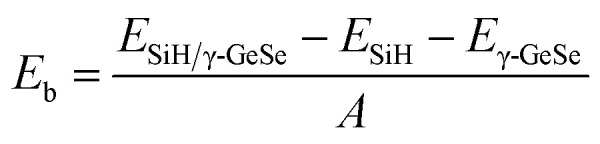
where *E*_SiH/γ-GeSe_, *E*_SiH_, and *E*_γ-GeSe_ represent the total energies of the heterostructure, the SiH monolayer, and the γ-GeSe monolayer, respectively, and *A* = *a*^2^ sin *α* is the area square of the heterostructure (*α* represents the angle between *a* and *b*.). The values of *E*_b_ for the SA1, SA2, SA3, SA4, SA5 and SA6 arrangements of the SiH/γ-GeSe heterostructure are −13.17, −13.62, −13.38, −8.52, −13.59 and −9.19 meV Å^−2^, respectively. These negative values indicate the stability of the heterostructures. Notably, SA2 arrangement exhibits the lowest value of the *E*_b_, specifying that this arrangement is the most favorable. Additionally, these values of the *E*_b_ are consistent with those observed in other typical van der Waals systems, such as SiH/InSe,^47^ SiH/CdCl_2_ (ref. 50) and C_3_N_4_/MoS_2_.^51^ This consistency suggests that the SiH and γ-GeSe layers are connected through weak van der Waals interactions. To further investigate the bonding characteristics between the SiH and γ-GeSe layers, we calculated the electron localization functions (ELF), using an ELF value of 0.5, which corresponds to the electron-gas-like pair probability.^52^ The 3D ELF of the SiH/γ-GeSe heterostructure in all six stacking arrangements is shown in Fig. 2. It is clear that electrons tend to accumulate between the Si and H atoms, as well as the Ge and Se atoms. This indicates that the Si and H atoms, along with the Ge and Se atoms, are bonded through covalent bonds. Furthermore, there is no significant electron accumulation between the SiH and γ-GeSe layers, suggesting a lack of direct bonding between these layers. This finding confirms that the SiH and γ-GeSe layers are connected through weak vdW interactions, which are essential for the future synthesis of such heterostructure.

**Fig. 3:**
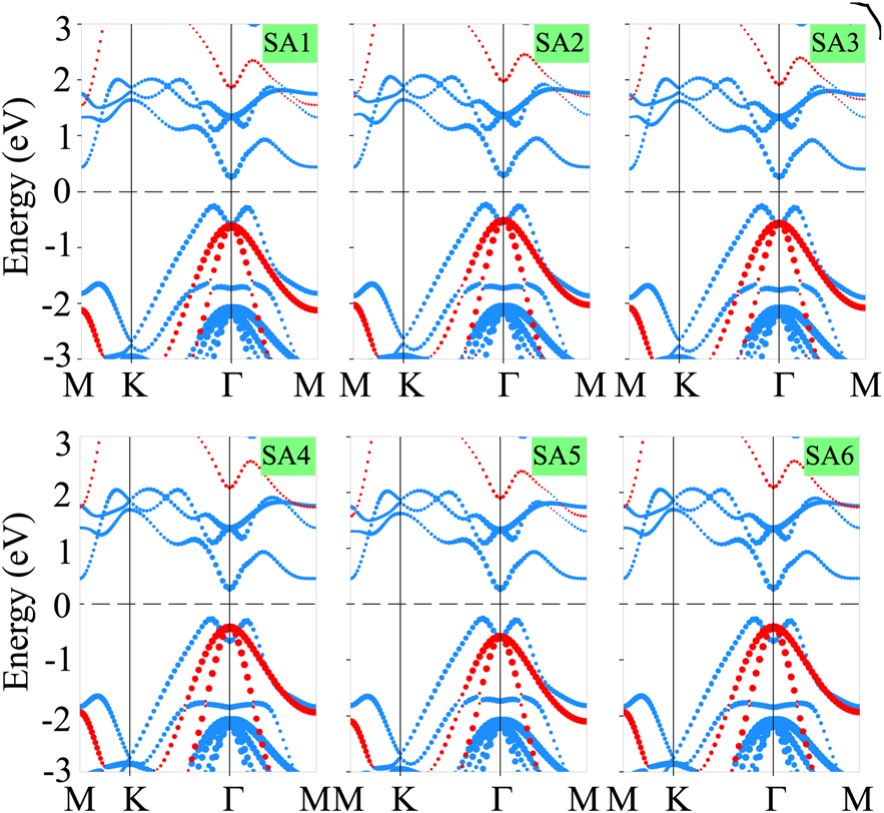
The weighted projections of the band structures for the SiH/γ-GeSe heterostructure in six different configurations. The SiH and γ-GeSe layers are represented by red and blue lines, respectively, to indicate their contributions.

Now, we consider the band structures of the SiH/γ-GeSe heterostructure, illustrated in Fig. 3 for all six arrangements. Our findings indicate that all six configurations display semiconducting behavior with indirect band gaps (in-*E*_g_) of approximately 0.53/0.93 eV for PBE/HSE method, which is narrower than that of the γ-GeSe monolayer (0.54/0.99 eV).^53,54^ Due to the reduced band gap, the heterostructure can absorb light more efficiently across a broader range of wavelengths. Furthermore, it is evident that the minima of the conduction band (CB) of the SiH/γ-GeSe heterostructure is located at the *Γ* point, while the maxima of the valence band (VB) lies along the K–*Γ* path. More interestingly, the VB and CB of the SiH/γ-GeSe heterostructure are dominated by contributions from the γ-GeSe layer. This indicates the formation of type-I band alignment (BA) across all six configurations. This means that the electrons and holes are confined within the same γ-GeSe material, leading to efficient recombination of charge carriers. This characteristic is highly advantageous for applications of the SiH/γ-GeSe heterostructure.

**Fig. 4:**
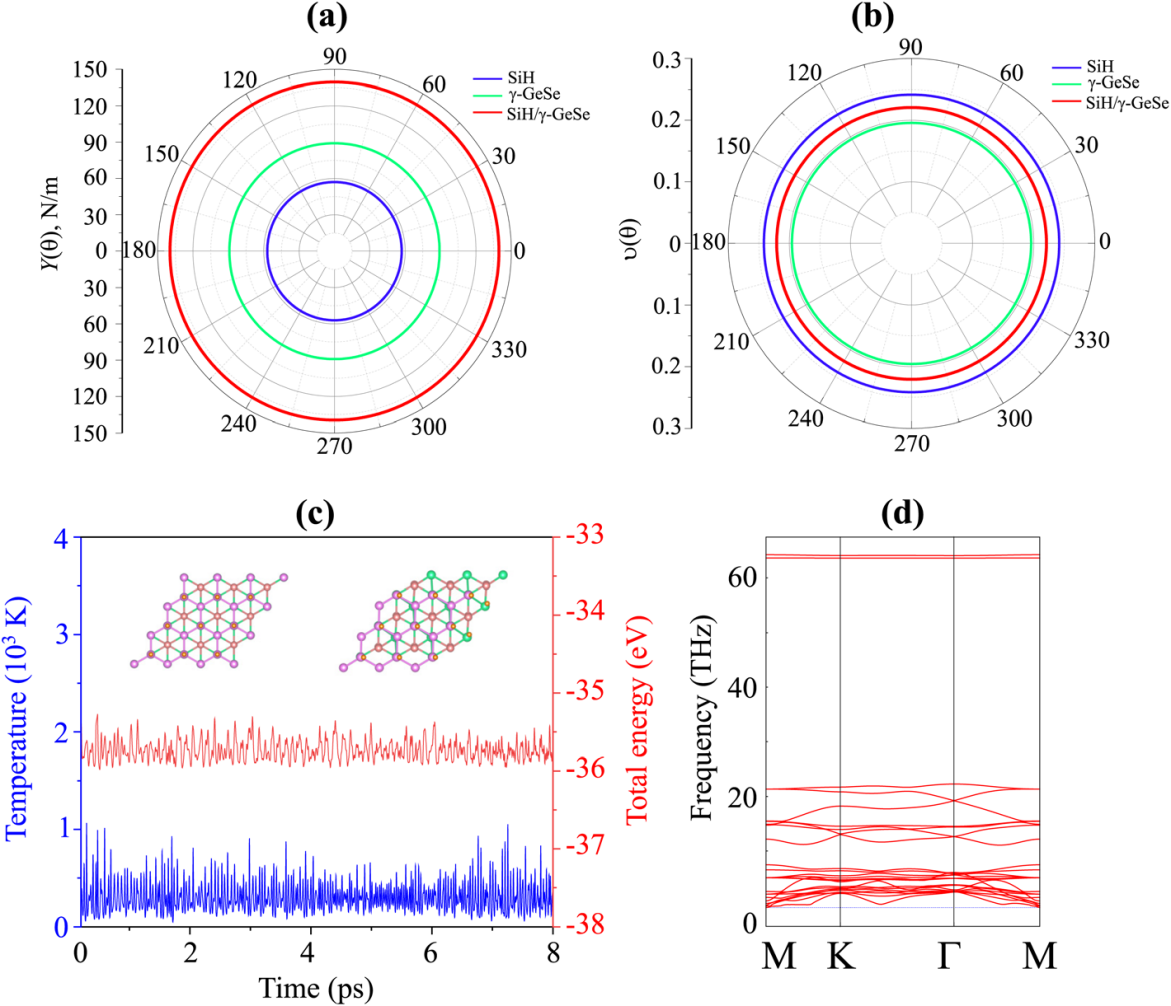
Angular dependence of the (a) Young modulus and (b) Poison ration of the SiH/γ-GeSe heterostructure. (c) The variations in the total energy and temperature and (d) the phonon spectra of the SiH/γ-GeSe heterostructure for the most favorable SA2 arrangement.

Furthermore, we assess the stability of the SiH/γ-GeSe heterostructure by performing the calculations on the mechanical behavior, AIMD simulation and phonon spectra. The elastic constants of the SiH/γ-GeSe heterostructure can be obtained by solving the following matrix:
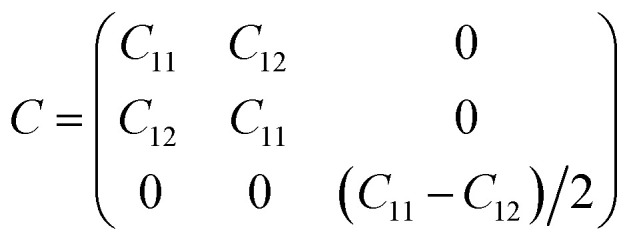


The SiH/γ-GeSe heterostructure exhibits only two independent elastic constants of *C*_11_ and *C*_12_, which are obtained to be 146.62 and 32.36 N m^−1^, respectively. The values of the elastic constants satisfy the Born–Huang criterion, indicating that the SiH/γ-GeSe heterostructure is mechanically stable. Furthermore, the Young modulus and Poison ration of the SiH/γ-GeSe heterostructure are also calculated as:2
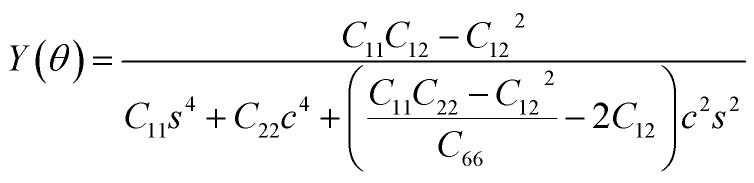
and3
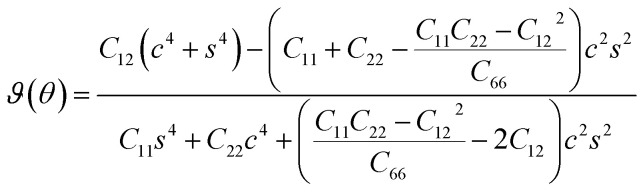
Here, *c* and *s* stand for the cos *θ* and sin *θ*, respectively. The dependence of the polar Young modulus and Poison ration of the SiH/γ-GeSe heterostructure on the angle are displayed in Fig. 4(a) and (b). The SiH/γ-GeSe heterostructure, along with its constituent SiH and γ-GeSe monolayers, exhibits isotropic mechanical properties. Moreover, our findings indicate a significant enhancement in the Young's modulus of the SiH/γ-GeSe heterostructure compared to its individual SiH and γ-GeSe layers. Specifically, the maximum Young's moduli of the SiH and γ-GeSe monolayers are measured to be 56.98 N m^−1^ and 89.11 N m^−1^, respectively. These values align well with those reported in previous studies.^17,55^ Remarkably, the Young's modulus of the SiH/γ-GeSe heterostructure is found to be enhanced to 139.47 N m^−1^, corresponding to the superposition of the moduli of the two monolayers. This enhancement indicates that the combined structure benefits from the mechanical properties of both constituent layers, showcasing the superior mechanical performance of this heterostructure. Additionally, AIMD simulations reveal that the total energy and temperature remain relatively stable, and no atomic distortions are observed (Fig. 4(c)). These findings show that the SiH/γ-GeSe heterostructure is thermally stable at the room temperature. Additionally, the phonon spectrum of the SiH/γ-GeSe heterostructure in the most favorable SA2 configuration reveals the absence of imaginary frequencies at the *Γ* point, confirming the heterostructure dynamical stability (Fig. 4(d)).

When the SiH/γ-GeSe heterostructure forms, charge redistribution occurs at the interface due to the difference in charge densities between the two materials. This redistribution can be analyzed through the charge density difference, which is defined as

**Fig. 5:**
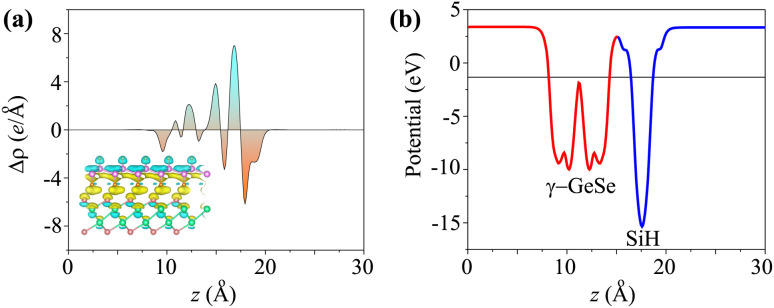
(a) The charge density difference and (b) electrostatic potential of the SiH/γ-GeSe heterostructure for the SA2 arrangement.

4 Δ*ρ*(*z*) = *ρ*(*z*)_SiH/γ-GeSe_ − *ρ*(*z*)_SiH_ − *ρ*(*z*)_γ-GeSe_

where, *ρ*(*z*)_SiH/γ-GeSe_ represents the charge density of the SiH/γ-GeSe heterostructure, *ρ*(*z*)_SiH_ and *ρ*(*z*)_γ-GeSe_ stands for the charge densities of the isolated SiH and γ-GeSe monolayers, respectively. A positive Δ*ρ* indicates an accumulation of electrons, whereas a negative Δ*ρ* signifies electron depletion. Upon the formation of the SiH/γ-GeSe heterostructure, charge redistribution occurs at the interface, where the SiH monolayer donates electrons to the γ-GeSe monolayer, as depicted in Fig. 5(a). The electrostatic potential further confirms this redistribution, showing that the potential of the SiH layer is more negative than that of the γ-GeSe layer, as illustrated in Fig. 5(b). The potential difference between the two layers is 6.38 eV, resulting in a built-in electric field directed from the γ-GeSe layer towards the SiH layer. This indicates electron migration from the SiH monolayer to the γ-GeSe monolayer.

Furthermore, it is evident that the optical absorption plays a crucial role in understanding the interaction of materials with light, providing valuable insights into their potential for applications in optoelectronics such as solar cells, photodetectors, and light-emitting devices. Hence, to analyze the optical properties of the SiH/γ-GeSe heterostructure, we calculated its optical absorption coefficient by deriving the real (*ε*_1_) and imaginary (*ε*_2_) parts of dielectric functions as follows:5
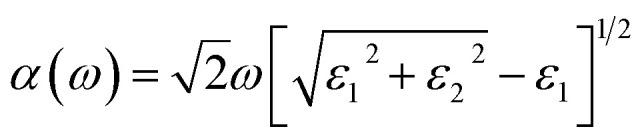


**Fig. 6:**
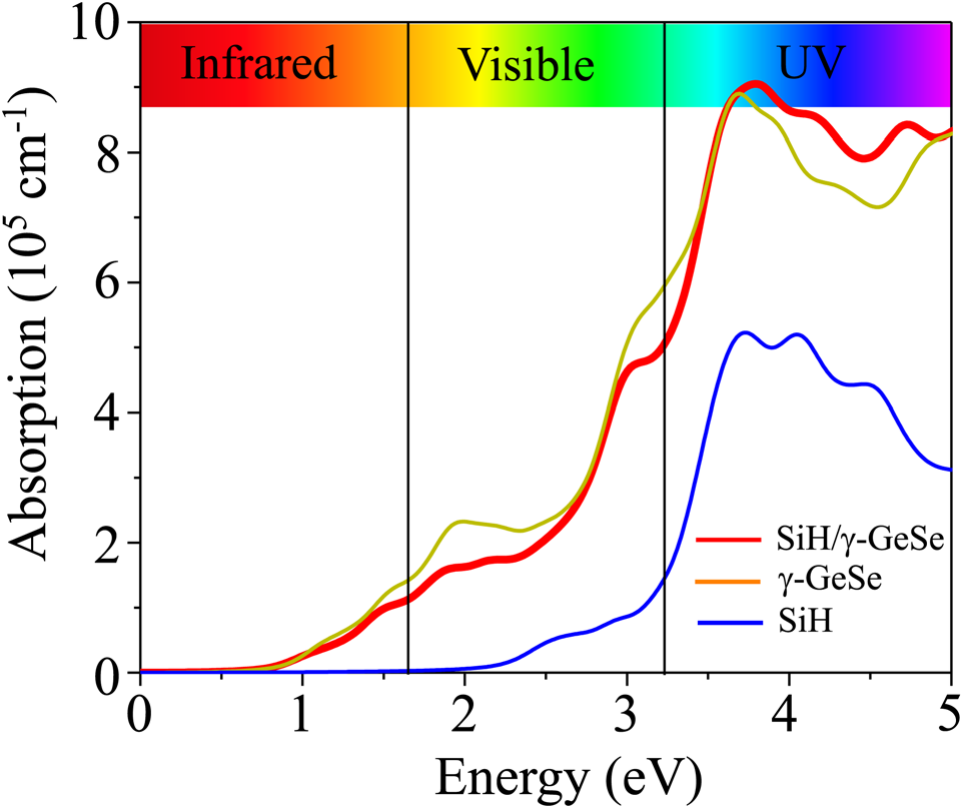
Optical absorption of the SiH, γ-GeSe monolayers and their SiH/γ-GeSe heterostructure.

The optical absorption of the SiH/γ-GeSe heterostructure is calculated using PBE method. Notably, it should be noted that the GW method for optical properties is widely recognized for providing better agreement with experimental values, as the band gap obtained from GW calculations is higher than that derived from the traditional PBE method.^56^ However, it is worth noting that the GW curve typically resembles the PBE calculated curve.^57^ Consequently, the PBE method can be considered a suitable approach for predicting the optical behavior of 2D materials. Compared to the isolated monolayers, the heterostructure demonstrates unique optical absorption features due to the coupling between the SiH and γ-GeSe layers, resulting in enhanced absorption in specific energy regions. The absorption coefficient of the SiH/γ-GeSe heterostructure is much close to that of the γ-GeSe layer, as displayed in Fig. 6. In addition, the absorption coefficient of the SiH/γ-GeSe heterostructure is slightly larger than that of the SiH layer in all region and higher than that of the γ-GeSe layer in the UV region. Notably, the absorption coefficient of the SiH/γ-GeSe heterostructure in the UV range reaches up to 9 × 10^5^ cm^−1^, which is greater than that of the SiH monolayer (5.2 × 10^5^ cm^−1^) and γ-GeSe monolayer (8.8 × 10^5^ cm^−1^). This enhancement in optical absorption highlights the potential of the SiH/γ-GeSe heterostructure for applications in optoelectronic devices. Additionally, the combination of these two materials not only broadens the absorption spectrum but also optimizes the efficiency of devices operating in the UV range. This versatility allows the SiH/γ-GeSe heterostructure to be used in a wide range of optoelectronic applications.

**Fig. 7:**
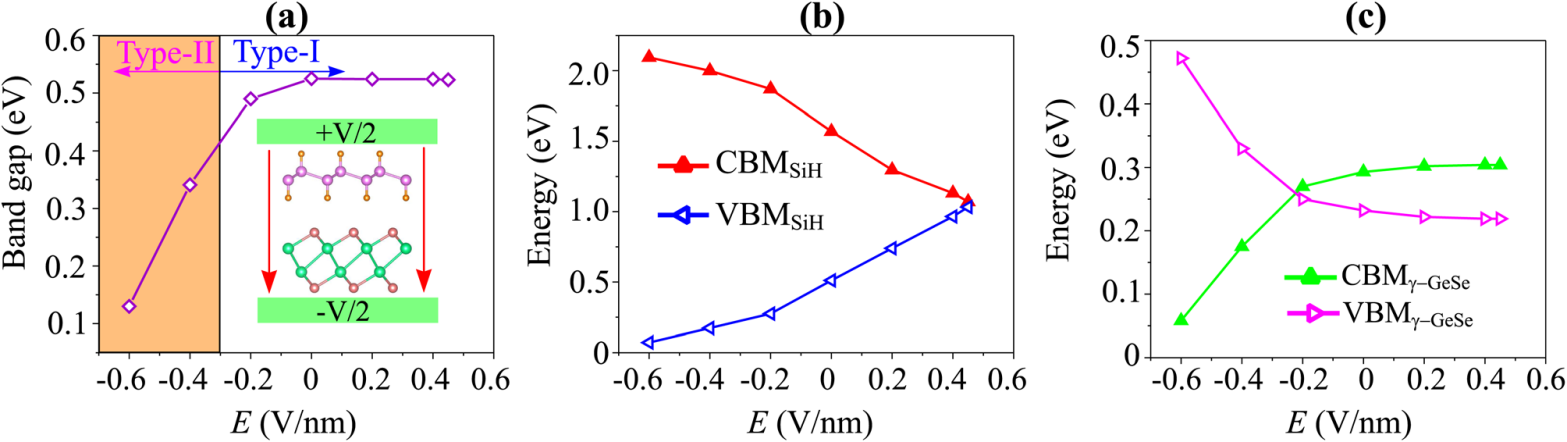
The variations in (a) band gap of the SiH/γ-GeSe heterostructure and (b) the band edges of SiH and (c) band edges of γ-GeSe layers.

Moreover, in a 2D van der Waals heterostructure, the application of an electric gating field (*E*) serves as a powerful method to adjust its electronic properties, such as band alignment and charge distribution, which significantly enhances device performance. Consequently, we investigate the effects of *E* on the electronic properties of the SiH/γ-GeSe heterostructure. The direction of the electric field *E* is considered positive when it points from the SiH layer towards the γ-GeSe layer. Fig. 7(a) illustrates how the band gap of the SiH/γ-GeSe heterostructure varies under the influence of an applied electric field. When a negative electric field is applied, the band gap decreases, whereas under a positive electric field, the band gap remains nearly unchanged. The mechanism behind this change can be explained as follows: the introduction of a negative electric field leads to a greater total electric field strength since the external and built-in electric fields are aligned in the same direction. This alignment enhances the potential difference across the SiH/γ-GeSe heterostructure, effectively reducing the band gap. In contrast, when a positive electric field is applied, it opposes the built-in electric field, weakening the total electric field. As a result, the opposing fields cancel each other out to some extent, leaving the band gap nearly unchanged.

Furthermore, to better describe the changes in the electronic properties of the SiH/γ-GeSe heterostructure, we analyze the variations in the band edges of both the SiH and γ-GeSe layers under the applied electric field, as shown in Fig. 7(b and c). It is evident that the VBM and CBM of the SiH layer shift in opposite directions as the applied electric field strength increases. As the electric field changes from −0.6 V nm^−1^ to +0.6 V nm^−1^, the VBM of the SiH layer rises while the CBM decreases. A similar behavior is observed in the γ-GeSe layer, where the band edges also shift in opposite directions. However, in this case, with increasing electric field strength from −0.6 V nm^−1^ to +0.6 V nm^−1^, the VBM decreases and the CBM increases. It's worth noting that the shifts in the band edges of the constituent SiH and γ-GeSe layers within the SiH/γ-GeSe heterostructure may lead to a switch in the band alignment. This dynamic adjustment can significantly affect the electronic properties, enhancing the versatility of the heterostructure for various applications.^58^ Furthermore, it should be pointed out that a large value of the applied electric field is generally restricted by the gate dielectric breakdown.^59^ However, such a high electric field strength can be achieved experimentally through the use of pulsed ac field technology, which allows for transient high-field applications.^60^

**Fig. 8:**
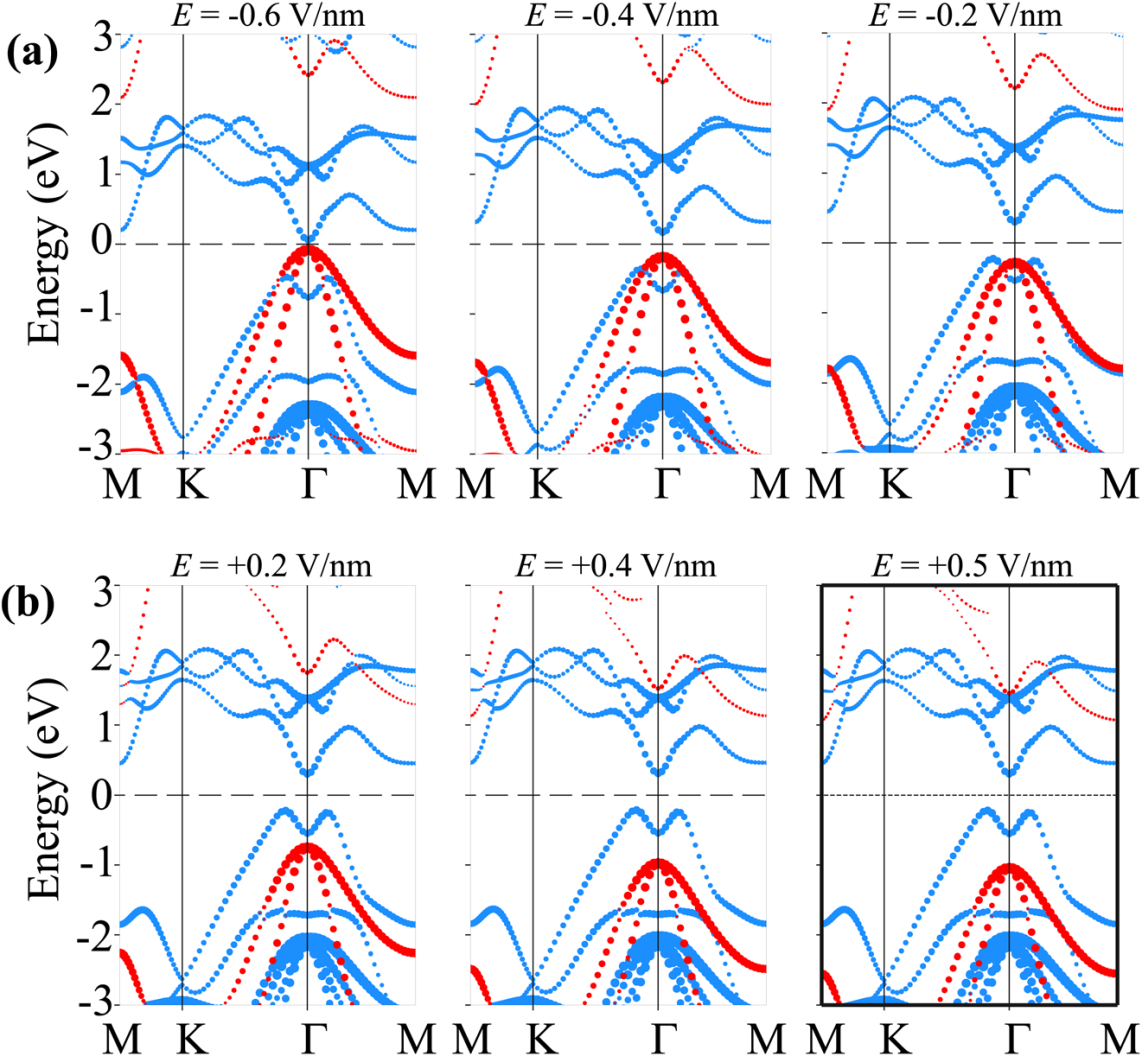
The weighted projections of the band structures of the SiH/γ-GeSe heterostructure under (a) negative-*E* and (b) positive-*E*.

Therefore, we further establish the weighted projections of the band structures of the SiH/γ-GeSe heterostructure under different electric fields, as shown in Fig. 8. It is evident that the application of a negative electric field facilitates electron transfer from the γ-GeSe layer to the SiH layer. This electron transfer induces a downward shift in the quasi-Fermi level of the SiH layer while raising the quasi-Fermi level of the γ-GeSe layer, which contributes to the decreased band gap of the SiH/γ-GeSe heterostructure. Moreover, the shifts in the quasi-Fermi level of the SiH and γ-GeSe layers trigger a transition from type-I to type-II band alignment at a specific magnitude of negative-*E*. As shown in Fig. 8(a), when a negative-*E* = −0.4 V nm^−1^ is applied, the VBM of the SiH/γ-GeSe heterostructure shifts from the K–*Γ* path (where it is located at *E* = 0 V nm^−1^) to the *Γ* point. This shift is primarily governed by the SiH layer, which dominates the VBM at the *Γ* point. At the same time, the CBM remains at the *Γ* point and is predominantly contributed by the γ-GeSe layer. More interestingly, further decrease in the negative-*E* to −0.6 V nm^−1^, both the VBM and CBM of the SiH/γ-GeSe heterostructure touch the Fermi level, resulting in the transition from semiconductor to metal. It should be noted that applying negative-*E* enhances total electric field strength because the external and built-in electric fields are aligned in the same direction. This alignment enhances the potential difference across the SiH/γ-GeSe heterostructure, effectively reducing the band gap. This change in the electronic structure results in two significant transitions: from an indirect to a direct band gap and from a type-I to a type-II band alignment within the SiH/γ-GeSe heterostructure. The transition to a direct band gap enhancing the potential for optoelectronic applications. Meanwhile, the shift to type-II alignment means that the electrons and holes are now spatially separated across the heterostructure, with electrons localized in the γ-GeSe layer and holes in the SiH layer, which could be beneficial for applications in photodetectors where charge separation is crucial. On the other hand, when the positive-*E* is applied, the overall electric field experienced by the heterostructure is reduced (Fig. 8(b)). As a result, the shift in the quasi-Fermi level of the γ-GeSe layer is minimal. Under these conditions, the SiH/γ-GeSe heterostructure retains its type-I band alignment and maintains an indirect band gap. The application of a positive-*E* stabilizes the original configuration of the heterostructure, making it more suitable for applications where charge confinement and minimized electron–hole separation are desired, such as in tunneling field-effect transistors (TFETs).

## 4 Conclusions

In conclusion, we employed first-principles calculations to investigate the electronic and optical properties of the SiH/γ-GeSe heterostructure and its remarkable tunability under applied electric fields. The heterostructure demonstrates stability, indicating its potential for future synthesis, and initially exhibits type-I band alignment accompanied by an indirect band gap. Optical absorption analysis reveals enhanced absorption in specific energy regions, underscoring its promise for optoelectronic applications. With the application of electric fields, the SiH/γ-GeSe heterostructure transitions to type-II band alignment and switches to a direct band gap, which enhances charge separation and light absorption. These findings highlight the versatility of the SiH/γ-GeSe heterostructure, positioning it as a strong candidate for a variety of electronic and optoelectronic applications.

## Conflicts of interest

There are no conflicts to declare.
